# Measuring subjective social status in children of diverse societies

**DOI:** 10.1371/journal.pone.0226550

**Published:** 2019-12-20

**Authors:** Dorsa Amir, Claudia Valeggia, Mahesh Srinivasan, Lawrence S. Sugiyama, Yarrow Dunham

**Affiliations:** 1 Yale University, Department of Anthropology, New Haven, CT, United States of America; 2 University of California, Department of Psychology, Berkeley, CA, United States of America; 3 University of Oregon, Department of Anthropology, Eugene, OR, United States of America; 4 Yale University, Department of Psychology, New Haven, CT, United States of America; University of Queensland, AUSTRALIA

## Abstract

Subjective Social Status (SSS) is a robust predictor of psychological and physiological outcomes, frequently measured as self-reported placement on the MacArthur Scale of Subjective Social Status. Despite its importance, however, there are still open questions regarding how early into ontogeny SSS can be measured, and how well SSS measures can be extended to non-Western and small-scale populations. Here, we investigate the internal consistency of responses to the MacArthur ladder across four cultures by comparing responses to more explicit social comparison questions. We conduct these comparisons among children and adolescents, ages 4 to 18, in India, the United States, and Argentina, in addition to those in two indigenous communities of the Ecuadorean Amazon marked by differing degrees of market integration (total N = 363). We find that responses are consistent in all populations, except for the more remote forager-horticulturalist Ecuadorian community. We also find that, consistent with findings among American adolescents, SSS declines with age. We then assess the test-retest reliability of the MacArthur Scale across two time-points: a subset of Indian participants (N = 43) within one week, and a larger, second sample of Indian participants after one year (N = 665). We find that responses are highly correlated within one week (*ρ* = 0.47), and moderately correlated after one year (*ρ* = 0.32). These results suggest that responses to the MacArthur ladder are internally consistent and reliable among children across a range of diverse populations, though care must be taken in utilizing these measures among children of non-industrial, small-scale societies.

## 1. Introduction

### 1.1 Subjective social status

A large body of literature has consistently documented the importance of subjective social status (SSS) as a predictor of psychological and physiological outcomes [1–7]. SSS is often used to describe “a person’s belief about his location in a status order” [8], or more broadly, “an individual’s perception of his or her place in the socioeconomic structure” [9]. In many cases, SSS explains variance above and beyond more objective measures of socioeconomic status, suggesting that perceptions of one’s position in the socioeconomic hierarchy have important implications for a broad swath of life outcomes, from depression to obesity to smoking [1,2,10,11]. This may be because subjective ratings capture more subtle aspects of social status not accessible through objective measures such as income and education [1,12], with the ability to better represent factors that underlie SES differences in behavior and health. For instance, SSS may better capture health-relevant consequences of perceiving a low status for oneself—such as chronic stress—than objective social status [12]. In line with this argument, several studies have shown that the correlation between objective and subjective measures of social status is moderate, suggesting that SSS measures are likely to independently predict outcomes [7,13,14]. Some have further suggested that self-reported SSS measures may actually be more valid measures of SES, as they summate across different indicators of objective status [1]. As subjective social status measures have the advantage of capturing subjectivity as a potential index of how the external realities of socioeconomic status are internalized, they may be better predictors of outcomes of interest for the same reason that income inequality is often a better predictor of well-being than absolute levels of income [15].

While SSS can be assessed in a multitude of ways, one of the most ubiquitous instruments deployed in the literature is the MacArthur Scale of Subjective Social Status (referred to here as the *MacArthur ladder*) [13,14]. This scale asks participants to rank their social standing on a 10-rung ladder, with those at the top frequently described as those who have the most money, the highest amount of schooling, and the jobs that bring the most respect [14]. Among the various versions, participants are asked to rank their family in comparison to reference groups such as other families in their society or community.

### 1.2. Subjective social status across culture and age

While the majority of research on SSS has been conducted among American populations, some have explored the role of SSS in predicting outcomes in other cultures. Researchers have deployed the MacArthur ladder to investigate SSS among adult participants in Taiwan [16], Germany [10], Japan [17], Brazil [18], China [19], and Mexico [20], documenting relationships similar to those found in American populations.

More recent work has also begun to explore subjective social status in early life. In 2001, an adolescent-friendly version of the MacArthur ladder was introduced, which asked students to rank their families compared to other families in their schools [14]. Goodman and colleagues demonstrated that this scale is both reliable and predictive, demonstrating intra-class correlation coefficients greater than 0.7, and robust predictions of physiological and psychological symptoms and conditions, such as depression and obesity [14]. Further, in a longitudinal study of American adolescents, Goodman and colleagues demonstrated that subjective social status tends to decline with age, stabilizing in older teenagers [21]. Additionally, among the same adolescents, lower reported standing on the MacArthur ladder predicted poor self-rated health, even after controlling for race and more objective measures of SES [21]. A recent meta-analysis of SSS supported these patterns, finding that SSS predicts health outcomes above and beyond objective measures of socioeconomic status among adolescents [9].

Integrating both culture and age, a third avenue of research has explored the influence of subjective social status on health and well-being among adolescents of diverse cultures. These studies have been conducted among young adults in Hungary [22], Mexico [23], Norway [24], Canada [25], Thailand [26], and Slovakia [27], among others. In the majority of these studies, researchers collected objective measures of social class—such as parental educational level—in addition to subjective social status through the MacArthur ladder and examined their relationship to largely physiological outcomes. In some cases, these studies have revealed different patterns than those found in the United States. Among Mexican adolescents, for instance, participants who perceived themselves as higher in subjective social status were *more* likely to report smoking or drinking behaviors [23]. A similar pattern was found among Slovakian girls, with higher SSS associated with greater smoking, while the pattern reversed for boys [27].

Despite these advances in assessing SSS cross-culturally and developmentally, a number of open questions remain. The first is whether or not measures of SSS can be extended earlier into development to measure subjective social status in early life. The second is how well these measures of SSS can be extended to non-Western and small-scale populations. In line with a major criticism of developmental psychology more generally [28], our current understanding of subjective social status is limited by researchers’ near-exclusive focus on individuals from Western, Educated, Industrialized, Rich, Democratic (WEIRD) societies [29]. Consequently, there is virtually no work on the development of SSS among small-scale populations, or on how broader sociocultural shifts, such as integration into Western market economies, might be related to perceived social status among children and young adults. Market integration—the degree to which one produces for, and consumes resources from the market [30], typically proxied through measures such as the percentage of calories purchased from markets [31]—is associated with rapid and significant changes across a broad range of traits related to well-being, such as cardiovascular disease and bone health [32–36]. A number of these traits have also been tied to measures of SSS in other populations [9,11,16,17,20,22,37]. As such, it may be useful to assess how SSS can be measured in these rapidly-changing, small-scale populations.

### 1.3. Participants and populations

In this study, we investigate the utility of measuring SSS using the MacArthur Scale of Subjective Social Status across children of four diverse cultures. Our sample includes participants from four countries: the United States, India, Argentina, and Ecuador. Below, we summarize key features of each culture. Some of these descriptions are reproduced from [38]. See the Supplement of Amir et al. 2019 for more details.

In the United States, we tested children in schools, museums, and the Yale Social Cognitive Development Laboratory in New Haven, CT. The city of New Haven is home to over 860,000 people, with approximately 18% of the population under the age of 15 [39]. New Haven’s economy is largely built around education and health care services, with Yale University and the Yale-New Haven Hospital functioning as the city’s largest employer. A recent Brookings study ranks New Haven as 6^th^ in a list of cities with the highest income inequality overlap [40], while a FiveThirtyEight analysis describes New Haven as the city that best represents the demography the United States as a whole [41].

In India, research was conducted in the city of Vadodara in the state of Gujarat, in Western India. Vadodara is the third largest city in the state, with a population of nearly 2 million people, about 9% under the age of 6 [42]. It is one of the more densely populated regions of the state, with an average population density of approximately 7000 persons / km^2^. It lies on major road and rail lines connecting the major cities of Mumbai, Delhi, and Ahmedabad. There is a large Hindu majority (approximately 85%) and a substantial Muslim minority (approximately 11%). Participants in this study were all students at a K-12 school, largely populated by children whose families earned less than $2000 per year [43]. The school is a private charitable institution that offers substantial financial aid to make it accessible to students from a variety of economic backgrounds.

In Argentina, research was conducted among the Toba/Qom community, an indigenous population belonging to the Guarycurúan linguistic family, from the Gran Chaco region [44]. Traditionally hunter-gatherers, the Toba/Qom participants in this study live in a peri-urban village near the city of Formosa, and rely on market goods for 90+% of their diet [45]. This research took place at a village known as Namqom, with a population of roughly 2,300 individuals across 100 hectares [46]. The Toba/Qom males of Namqom participate primarily in wage labor, which forms the basis of the household economy [47]. Women partake mostly in childcare, homemaking, and basket weaving, with some employed as cooks, housemaids, or teaching assistants in the nearby city of Formosa [47]. Its proximity to urban centers has afforded Formosa access to resources such as electricity, roads, and government food subsidies [48]. Less than 10% of the Toba diet comes from non-market goods [45]. For descriptive purposes, the communities in India, Argentina, and the United States are classified as “High” in market integration as the vast majority of calories comes from market goods.

In Ecuador, we worked in the Morona Santiago Province with the Shuar, a large indigenous Amazonian population of southeastern Ecuadorian extending into northeastern Peru. Traditionally, the Shuar economy was based on horticulture, hunting and fishing. Nuclear family households or household clusters were semi-mobile, coming together for collaborative action such as warfare (Harner 1984; Karsten 1935). Homes were constructed of palmwood or cane (*wadua*) walls, palm thatched roofs and dirt floors, with cooking fires maintained inside the house. In the mid-1960s, Shuar began to aggregate into more centralized communities or centers (*centros*) in response to mission and colonial forces (Salazar 1977; Rubenstein 2001).

Over the last two decades, the Shuar have experienced increasingly rapid, but highly differential rates of market integration [32]. Most Shuar in the study region now reside in permanent communities, comprised primarily of a few older individuals, their descendants and affines, with households centered around open community spaces and/or sports fields. The nuclear family household remains the primary economic unit, and individualism remains an important part of the Shuar ethos, with a strong cultural norm toward individual decision-making [49]. Most Shuar homes in the study area are constructed of rough chainsaw-cut lumber with tin roofs and wooden floors, although some more traditional structures remain [38]. Some houses have an adjacent traditionally constructed kitchen or cooking area. In others, cooking is done on a propane gas stove (*cocineta*). Chickens, dogs, and other animals often enter houses.

We worked with Shuar in two regions, separated by the rugged, sparsely populated Cordillera de Cutucú: the eastern cross-Cutucú region and the western Upano Valley region. In both regions, the majority of calories still come from traditional horticultural products, primarily manioc (*manihot esculenta*), plantains (*musa sp*), papa china (*colocasia esculenta*), and yams (*Dioscorea trifida*), augmented by a variety of seasonal fruits and insects, and domesticated poultry and eggs. Fish and game are more important cross-Cutucú. Households are relatively large, averaging 6.85 in the Upano Valley and 7.11 in the cross-Cutucú region, with 53% of the population under 15 years of age [32]; Blackwell et al. 2010). In both regions, income comes primarily via occasional sales of produce, animals, timber or other forest products. Produce sales are limited in the cross-Cutucú due to difficulty of transport. Some people engage in occasional day wage labor, again less common trans-Cutucu where people have to move to other regions for work [32,49].

In sum, Shuar in the cross-Cutucú region primarily rely on horticulture, fishing and hunting for subsistence [33]. Ethnographic and food frequency data suggest that market foods play only a minor role in the diet, with any market food item being consumed in a household only about 4 times per week [32]. We have therefore classified these communities as “Low” in market integration. In contrast, Shuar in the Upano Valley region live closer to regional centers and have greater participation in the market economy, with significantly higher frequency of a market food item being consumed in a household per week, 17, than the cross-Cutucú Shuar [32]. Based on these ethnographic and quantitative measures of market integration in these communities [32,33], we have classified Upano Valley communities as “Medium” on the market integration spectrum.

We first investigate whether participants’ responses to the MacArthur ladder are consistent with a set of explicit follow-up questions that ask participants to make social comparisons. We consider the relationship between these social comparison questions with the MacArthur ladder both individual and in aggregate. We then assess the test-retest reliability of MacArthur ladder responses in two samples of Indian youth at two time-points, asking children to respond to the same question within one week and one year of their previous responses. A summary of participants can be found in **Table 1.** Age distributions across all populations can be found in **Table 2**. Study methods were approved by the institutional review boards of Yale University. In Ecuador, additional approval came from community leaders and the Federación Interprovincial de Centros Shuar (FICSH). As outlined in our IRB protocol, we obtained consent from parents or guardians in order for their children to participate. In the United States and India, this was through written consent. In Ecuador and Argentina, this was through verbal consent, gathered and witness by certified research personnel.

**Table 1:** Descriptive summary of all participants.

Country	Population	Economy	Market Integration	N (males)	Mean age (range)
**Argentina**	Toba/Qom	Wage labor	High	69 (31 male)	10.7 (4–18)
**Ecuador**	Cross-Cutucú Shuar	Horticulture, fishing, hunting, gathering, limited agro-pastoralism and sporadic wage labor	Low	58 (31 male)	10.8 (5–17)
**Ecuador**	Upano Valley Shuar	Horticulture, fishing, hunting, gathering, limited agro-pastoralism and sporadic wage labor	Medium	81 (43 male)	10.6 (5–17)
**India, Sample 1**	Vadodara	Professional, trade/service, labor	High	86 (43 male)	9.8 (6–14)
**India, Sample 2**	Vadodara	Professional, trade/service, labor	High	665 (387 male)	13.1 (6–17)
**USA**	New Haven	Professional, trade/service, labor	High	69 (31 male)	8.9 (4–15)
			*Total*	*1*,*028 (566)*	*10*.*7 (4–18)*

**Table 2:** Number of participants by age, across the full sample.

**Age:**	4	5	6	7	8	9	10	11	12	13	14	15	16	17	18
***N*:**	*4*	*14*	*24*	*37*	*46*	*48*	*43*	*140*	*165*	*172*	*146*	*112*	*44*	*9*	*5*

## 2. Methods

**Fig 1:**
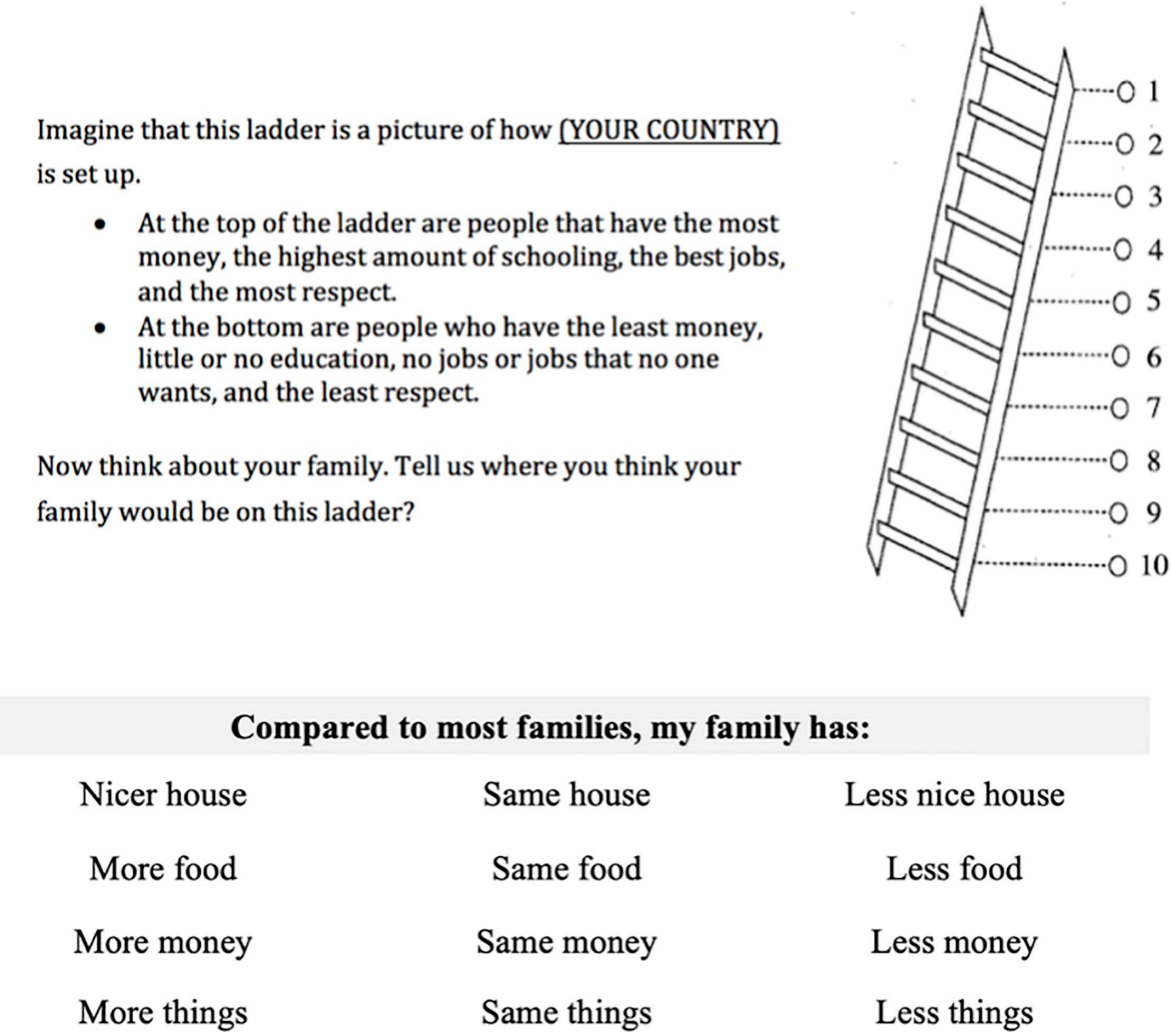
The MacArthur Ladder and the social comparison questions presented to participants.

We used the MacArthur Scale of Subjective Social Status to assess SSS, utilizing a version in which the reference groups were other families in the same country. We then asked four follow-up questions that asked participants to make a series of social comparisons. Experimenters verbally walked the participants through a worksheet with the illustration & questions in **Fig 1**. The survey was conducted in English in the United States and India and was translated by native speakers of Spanish for Ecuador and Argentina. In Ecuador, participants were recruited at community events, and tested in a private hut by the lead researcher. In Argentina, participants were visited at their homes by research assistants from the larger Chaco Area Reproductive Ecology (CARE) research initiative. In the United States, participants were tested in the Social Cognitive Development lab at Yale University, in addition to testing in private testing rooms in schools, and in semi-private testing stations in museums. In India, children were tested in private testing rooms in their school. In the India site, English is the primary language of instruction, but the lead researcher also collaborated with a language interpreter to ensure instructions were understood in both Hindi and Gujarati.

To test whether responses on the MacArthur ladder were consistent with more explicit social comparison questions, we summed across the four comparison questions to create a Social Comparison Index (SCI). Responses indicating participants viewed their family as having *more* of something (nicer house, more money, etc.) were coded as a 1, having the *same* as other families was coded as a 2, and having *less* was coded as a 3. The Social Comparison Index is the sum of responses across the four questions. Higher scores on the Social Comparison Index therefore indicate the participant views their family as having less than other families. Responses to the MacArthur ladder were coded in the same direction; that is, a response of 1 indicates a high perceived social status, while a response of 10 indicates a low perceived social status. This portion of the investigation—the MacArthur ladder and Social Comparison questions—always followed two short games measuring children’s preferences in a delay discounting and risk aversion task (Amir et al., 2019).

Next, to measure the test-retest reliability of the MacArthur ladder, we assessed the correlation between answers in two samples of children in India. In the first sample (N = 43), we calculated the correlation of responses to the ladder within one week of the original question. In a larger second sample (N = 665), we calculated the correlation of responses to the ladder approximately one year after the original question. The SSS question in this sample was identical to the original sample, and was administered as part of a larger study through UC Berkeley and Yale focused on social cognitive development.

## 3. Results

### 3.1. How consistent are MacArthur ladder responses with more explicit social comparison responses?

**Fig 2:**
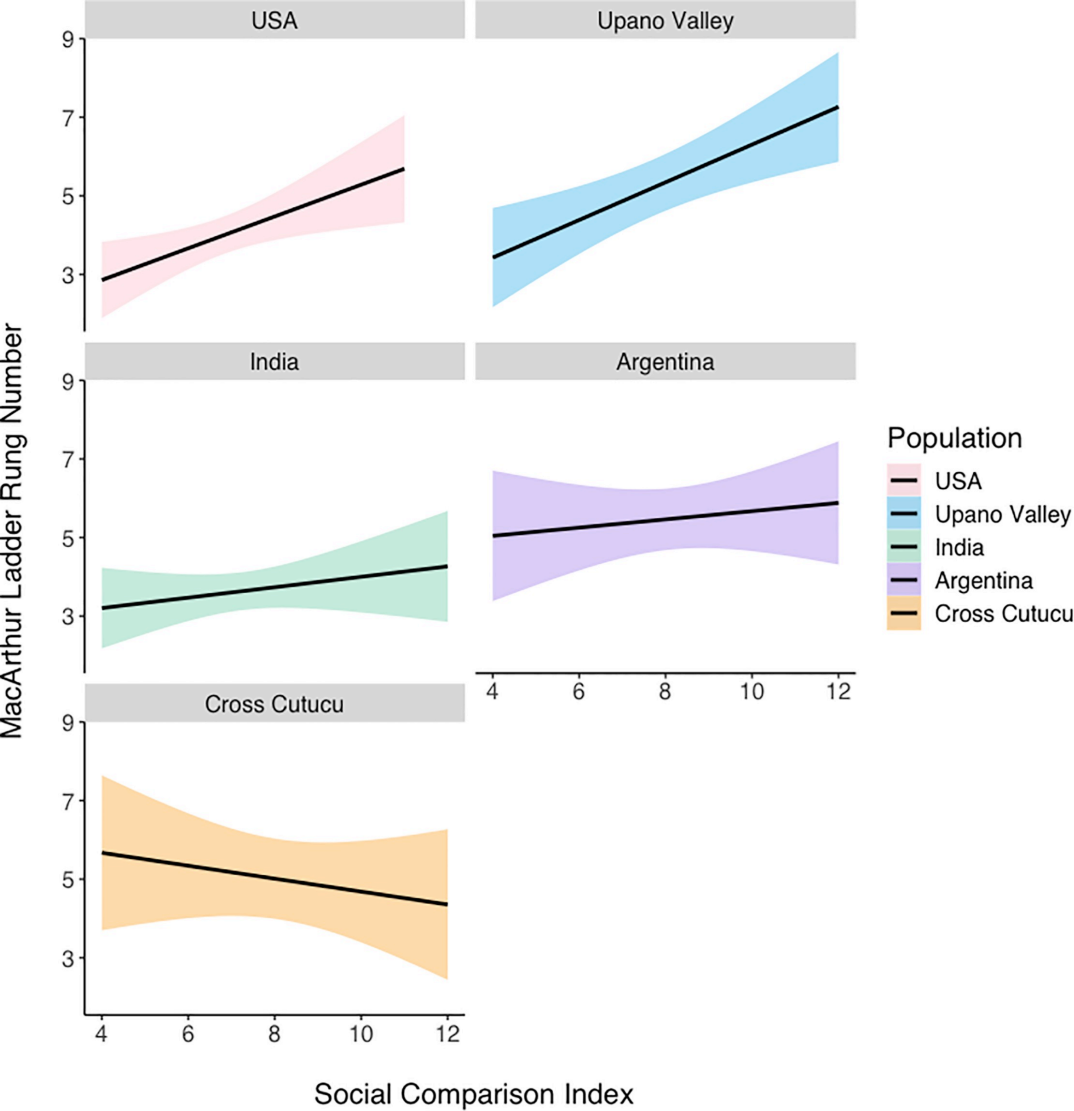
The relationship between Social Comparison Index score and responses to the MacArthur ladder across populations. Lines are linear regression lines. Shaded regions represent 95% confidence intervals.

**Fig 3:**
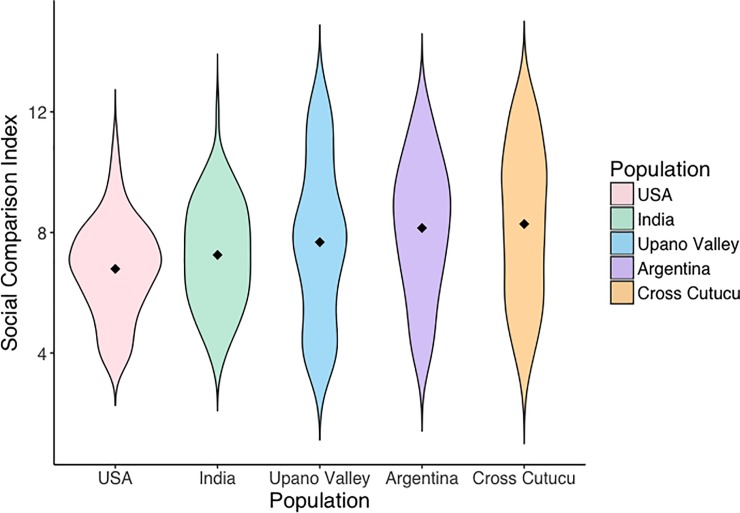
Violin plots demonstrating the distributions of Social Comparison Index scores across regions. The outlines illustrate kernel probability densities at each given score. The black diamonds indicate mean scores.

We first tested the consistency of responses to the MacArthur ladder with the responses to the social comparison questions. We found that the Social Comparison Index is positively predictive of MacArthur ladder responses (β = 0.211, SE = 0.073, p = 0.004) (see **Table 3**), suggesting that responses on the two measures are generally consistent. Importantly, however, we also found an interaction between region and Social Comparison Index scores, such that children in cross-Cutucú Ecuador showed a slightly *negative* relationship between MacArthur ladder responses and Social Comparison Index scores (β = -0.569, SE = 0.274, p = 0.039). This interaction is visualized in **Fig 2,** which depicts the relationship between responses on the MacArthur ladder and Social Comparison Index scores across populations. Violin plots demonstrating the means and distributions of Social Comparison Index scores across regions can be found in **Fig 3**.

**Table 3:** Effects and standard errors from linear models predicting ladder placement in the full sample (Model 1), the full sample with interactions (Model 2). The baseline for region is the United States. Table also shows goodness of fit.

	Model 1	Model 2
(Intercept)	2.55***	1.24
	(0.60)	(1.54)
Social Comparison Index (SCI)	0.21**	0.40
	(0.07)	(0.22)
India	-0.45	1.44
	(0.47)	(2.03)
Upano Valley Ecuador	1.03*	0.27
	(0.48)	(1.85)
Argentina	1.21*	3.39
	(0.50)	(2.03)
Cross-Cutucú Ecuador	0.74	5.09*
	(0.54)	(2.06)
SCI * India		-0.27
		(0.28)
SCI * Upano Valley		0.08
		(0.25)
SCI * Argentina		-0.30
		(0.27)
SCI * Cross-Cutucú		-0.57*
		(0.27)
R^2^	0.09	0.12
Adj. R^2^	0.07	0.09
Num. obs.	346	346
RMSE	2.86	2.83

***p < 0.001

**p < 0.01

*p < 0.05

Statistical models

### 3.2. How do responses to the MacArthur ladder vary across age?

To examine the effects of age, we built a multiple regression model with age, country, and Social Comparison Index score as predictors (**Table 4**).

**Table 4:** Effects and standard errors from a linear model predicting ladder placement with age as a predictor (Model 3) and the interaction between age and country (Model 4). The baseline for region is the United States. Table also shows goodness of fit.

	Model 3	Model 4
(Intercept)	1.26	1.36
	(0.74)	(1.31)
Social Comparison Index (SCI)	0.20**	0.20**
	(0.07)	(0.07)
India	-0.58	-0.89
	(0.47)	(1.79)
Upano Valley Ecuador	0.70	0.83
	(0.49)	(1.70)
Argentina	0.92	0.46
	(0.51)	(1.62)
Cross-Cutucú Ecuador	0.39	0.76
	(0.55)	(1.97)
Age	0.16**	0.15
	(0.05)	(0.13)
India * Age		0.03
		(0.19)
Upano Valley Ecuador * Age		-0.01
		(0.17)
Argentina * Age		0.04
		(0.16)
Cross-Cutucú Ecuador * Age		-0.03
		(0.19)
R^2^	0.11	0.11
Adj. R^2^	0.09	0.08
Num. obs.	344	344
RMSE	2.83	2.85

***p < 0.001

**p < 0.01

*p < 0.05

**Fig 4:**
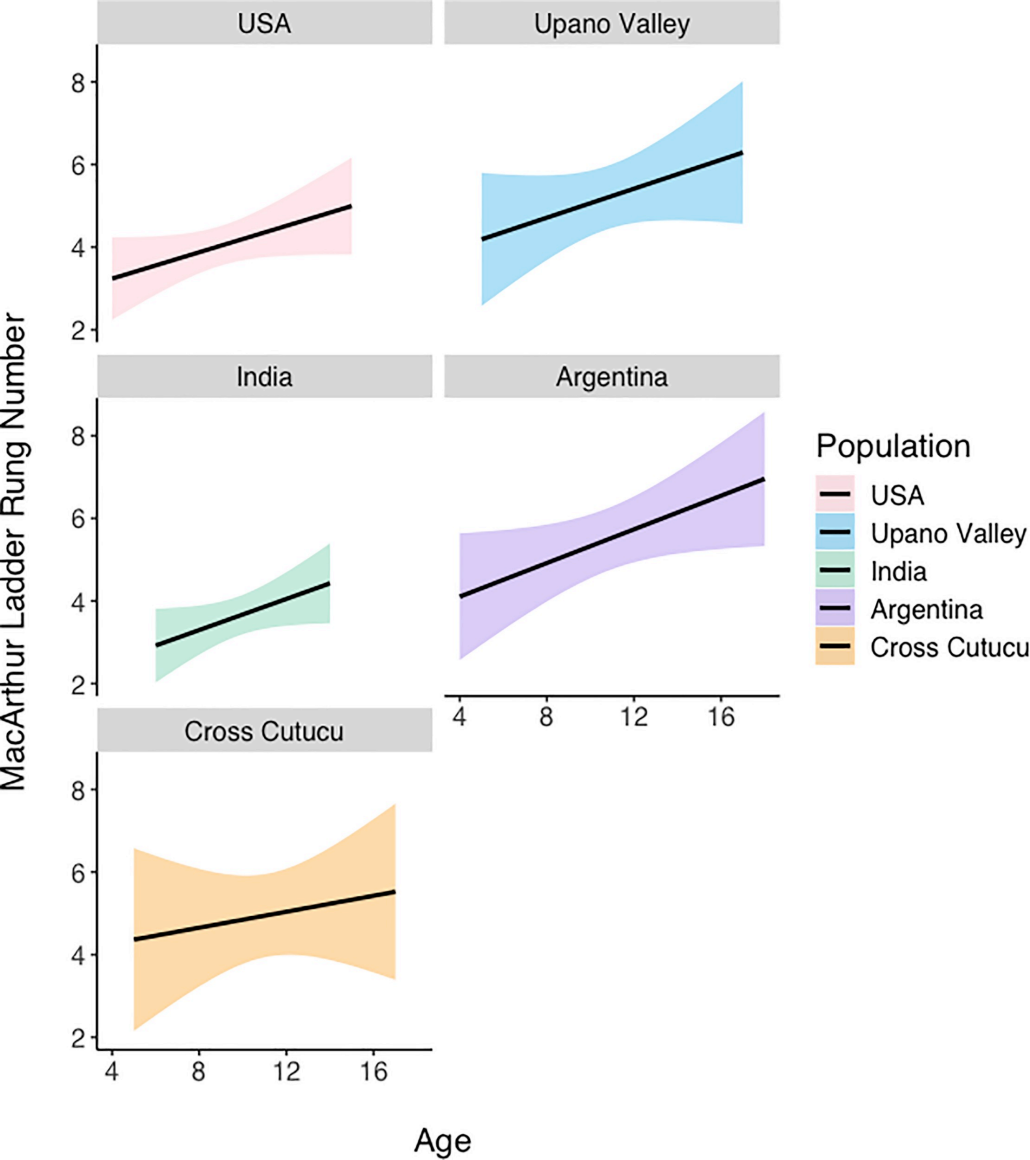
MacArthur ladder responses by age, across populations. Lines are linear regression lines. Shaded regions represent 95% confidence intervals.

We find that age is significantly predictive of responses to the MacArthur ladder, such that as children get older, they are more likely to rank themselves on a lower rung of the ladder (β = 0.157, SE = 0.051, p = 0.002) (**Fig 4**). The Social Comparison Index remains predictive of MacArthur ladder responses after controlling for age (β = 0.0196, SE = 0.072, p = 0.007), as does the slightly negative relationship between Social Comparison responses and MacArthur ladder scores among cross-Cutucú Shuar children, though this is now trending (β = -0.520, SE = 0.273, p = 0.058). We do not find an interaction between age and populations (ps > 0.78).

### 3.3. How do individual social comparison questions relate to SSS ladder scores?

**Fig 5:**
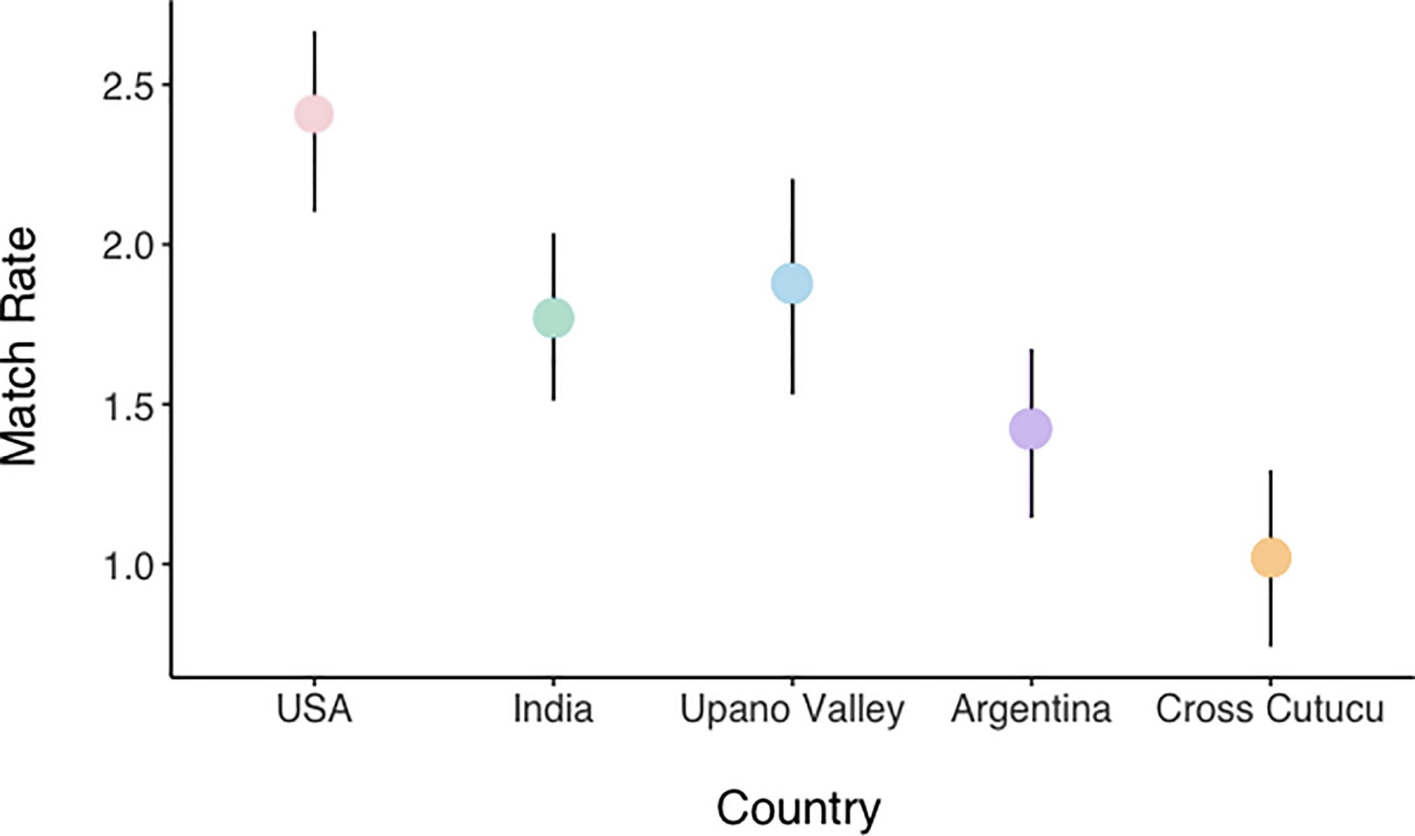
Match rates between individual social comparison scores and ladder response, by country. Error bars represent bootstrapped 95% confidence intervals.

We next turned to an item-specific assessment of the relationship between social comparison responses and the SSS Ladder. First, we computed “match rates” between each item in the SCI and the ladder. For ease of comparison between the two indices, we considered rungs 1–3 on the SSS Ladder to be conceptually equivalent to “high/more than” in the SCI, rungs 4–7 as “the same”, and rungs 8–19 as “low/less than”. We then computed a match rate—the sum of the total number of matches across the four items—to see how similar responses were on the two scales. We find, in line with the analyses above, that the strength of the match varies by site with match rates highest in the United States and lowest in cross-Cutucú Ecuador (**Fig 5**).

Next, we disaggregated the items of the Social Comparison Index and explored how each item related to ladder responses. While the reliability of the four items was acceptable across the full data set (Cronbach’s alpha = 0.7), we uncovered variation across sites, with reliability falling somewhat below desirable ranges in some sites, most notably the USA (see Table 5).

**Table 5:** Standardized Cronbach’s alphas for the Social Comparison items across regions.

Region:	Overall	Argentina	Cross-Cutucú Ecuador	India	Upano Valley Ecuador	USA
Standardized alpha:	0.70	0.70	0.67	0.59	0.79	0.53

**Fig 6:**
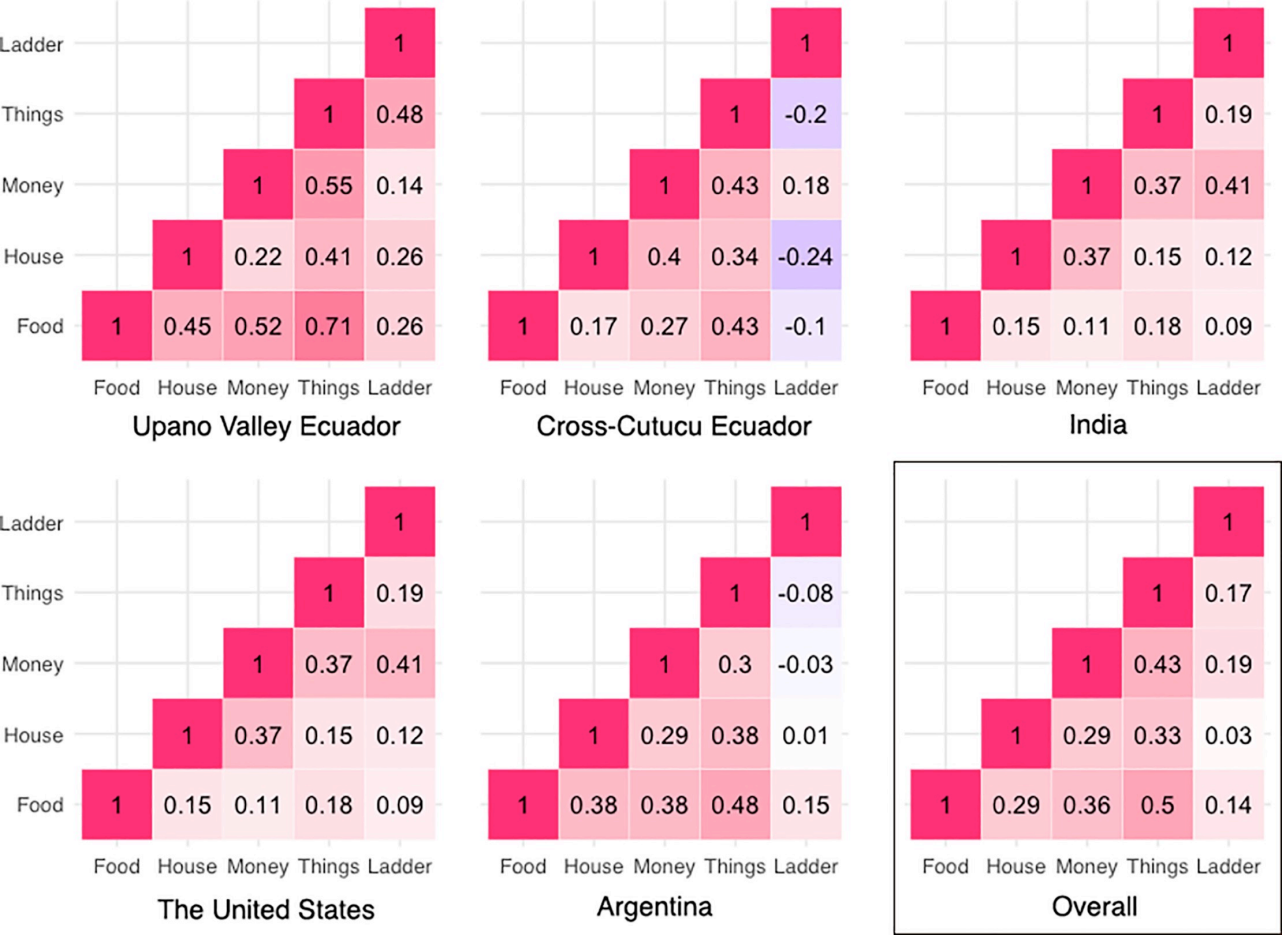
Pearson correlations between each of the social comparison items to one another and to the SSS ladder response across all regions, separately and pooled.

To do this, we built a correlation matrix to examine the relationship between each of the social comparison items with one another and with SSS ladder score (**Fig 6**). When looking at pooled data from all sites, comparisons of money, number of things, and amount of food are more strongly correlated with ladder scores than type of house. There is, however, substantial regional variability. In India, the United States, and cross-Cutucú Ecuador, the money comparison question is most strongly related to ladder responses. In Argentina, the most predictive item is amount of food, and in the Upano Valley, the most predictive item is number of things.

### 3.4. How consistent are responses on the MacArthur ladder across one week and one year?

**Fig 7:**
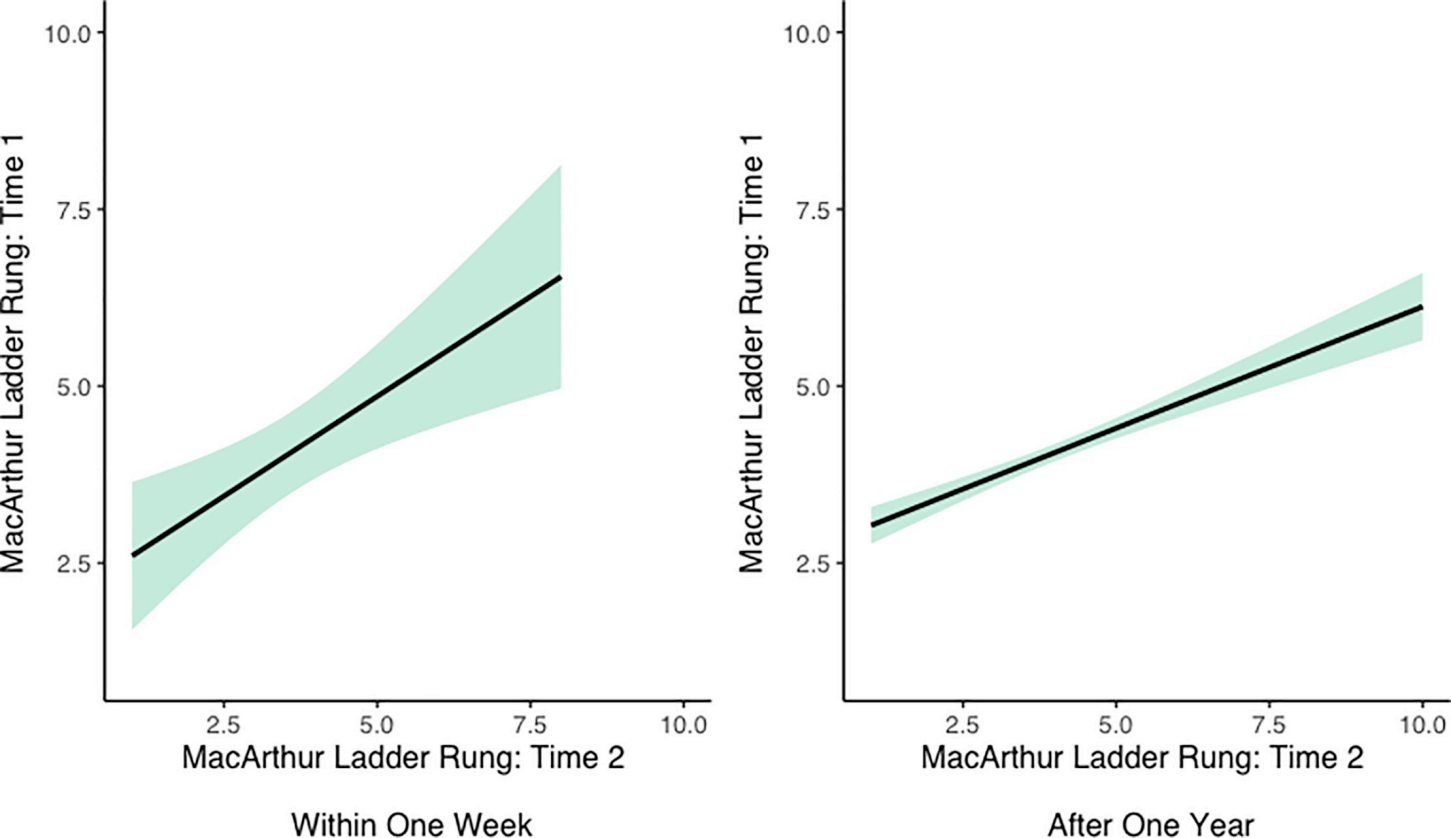
Correlation of responses to the MacArthur ladder within one week and after one year of original question among Indian participants. Lines are linear regression lines. Shaded regions represent 95% confidence intervals.

We next assessed the correlation between MacArthur ladder responses among a subset of Indian children (N = 43) who responded to the ladder measure for a second time within one week of their original response. The Pearson’s product-moment correlation between these two responses was significant (*ρ* = 0.469, p = 0.002), showing that responses were highly correlated within one week. We then measured this same correlation among a larger sample of Indian youth (N = 665) after one year. This relationship was also significant (*ρ* = 0.324, p < 0.0001), though less strongly correlated. The correlations between first and second responses across both time points can be found in **Fig 7**. We also conducted a paired t-test to evaluate whether children placed themselves lower on the ladder upon retest after one year. There was no significant difference between ladder placement in the initial testing (M = 4.06, SD = 1.74) and the re-testing one year later (M = 3.99, SD = 1.65); t(664) = 0.924, p = 0.356.

## 4. Discussion

Given the important role of SSS in predicting physiological and behavioral outcomes across cultures, we set out to test the internal consistency and test-retest reliability of the MacArthur ladder among children from four diverse societies. We first assessed the internal consistency of responses to the MacArthur ladder by comparing them to a series of explicit social comparison questions. We find that, in general, participants in these cultures respond to the MacArthur ladder in ways that are consistent with their answers to follow-up social comparison questions. However, we also find that the strength of this relationship varies across cultures, with the weakest correlation in Argentina. This relationship is also reversed among Shuar children in cross-Cutucú Ecuador. There are a number of possible reasons for this inconsistency. One may be due to the fact that cross-Cutucú Shuar children differ significantly from children in the United States, India, and Argentina as they live in an unindustrialized, rainforest ecology, in communities where members still forage, hunt, fish, and cultivate their own foods. Due to their remote location—only accessible by canoe—it’s likely that many cross-Cutucú Shuar children have not ventured far from their home communities. This can help explain why their responses on the MacArthur ladder are not consistent with their responses to social comparison follow-up questions: it may be difficult for them to rank their family in comparison to other families in Ecuador if they have had limited exposure to other families. This hypothesis is consistent with the pattern we observed among Shuar children in the Upano Valley region of Ecuador, who give consistent responses to the MacArthur ladder and social comparison questions and behave more like American children than cross-Cutucú Shuar children. The Shuar children of the Upano Valley live in communities marked by a similar ecology & culture when compared to their cross-Cutucú counterparts but are more likely to have exposure to other families, because they live near national highways and are neighbors to a larger number of non-Shuar families. Children in these regions also periodically accompany adults into larger town centers for market days. The relative weight of these factors such as market integration and admixture should be assessed further. Additionally, children across all sites may be interpreting the descriptor ‘family’ differently. While the nuclear family does comprise the core unit of family composition across all of the sites, it is certainly possible that children may have been considering other units such as the extended family when making judgements. Overall, our findings suggest that the MacArthur ladder is more appropriate for participants in market-integrated populations, and should be used cautiously in subsistence-based, small-scale societies.

When considering each item in the Social Comparison Index individually, we find that a participant’s judgment of how much money their family has is most strongly related to ladder placement. However, when breaking down item-level correlations by country, we find variation across regions as to which item is most strongly related to ladder placement. In the United States, India, and cross-Cutucú Ecuador, money remains the strongest item, but in Argentina and Ecuador, the strongest items are food and number of things, respectively. These results suggest that how children conceive of social status and what items are most influential in those conceptions are variable across cultures. They also underscore the importance of cross-cultural work when trying to understand subjective social status; the behavior of American children cannot be generalized to other populations.

Consistent with previous findings in the literature demonstrating that subjective social status declines with age prior to stabilizing in older adolescence [21], we find age to be a strong predictor of MacArthur ladder responses. Across all populations, we find that children are more likely to place themselves lower on the MacArthur ladder as they age. We also find that the Social Comparison Index is still predictive of MacArthur ladder scores, even after controlling for age. This suggests that across populations and stretching back to younger ages, participants are responding in consistent ways to both the ladder and social comparison questions. However, even after controlling for age, we still find some support for the inconsistent nature of cross-Cutucú children’s responses to the ladder and follow-up questions, which remain slightly negatively correlated.

We also assessed the test-retest reliability of MacArthur ladder scores among two samples of Indian children at two time points: within one week, and after one year. We find that responses are significantly correlated across these two time-points (*ρ* = 0.47 and *ρ* = 0.32, respectively), suggesting that this measure is reliable. These correlations, while moderate, are comparable to the within-person correlations found by Goodman and colleagues (which were 0.35; [21], suggesting the test-retest reliability of the MacArthur ladder is similar in both the United States and India. These results further suggest that comparable consistency in responses can be found in samples younger than those found in the original Goodman et al. paper, as the average age of Indian participants in our study was lower than the average age of American participants in the Goodman sample (which centered around 14.4 years; [21].

The observed inconsistencies between ladder and social comparison responses raise questions about the cross-cultural applicability of the MacArthur ladder. It’s possible that ladder responses don’t line up with social comparison questions because the ladder conflates many facets of social comparison. It may be that possessions such as houses, food items, money, and belongings don’t track each other to same extent across societies, potentially explaining the variation we observe across cultures. And as we observed in the item-level analyses, they differentially relate to subjective social status judgments across regions. Conversely, it’s also possible that other variables, not included in our social comparison questions, vary more closely with perceived social status in these diverse cultures. As this is the first study to assess SSS among children of small-scale populations, further work is required, ideally in conjunction with ethnographic observations and semi-structured interview, to better understand how social status is perceived in small-scale societies.

There are a number of limitations to this study, along with avenues for further research. The first and arguably most important limitation in this manuscript is that we lack the data to formally validate children’s responses by linking them to more objective measures of social status (e.g. household income) or other subjective measures (e.g. their parents’ responses to the same question). In previous work, the basic structure of the MacArthur ladder has been validated for use in youth populations [14] through comparisons of participants’ answers with their parents. However, to date, these validation efforts have not extended to non-WEIRD societies where standard measures—objective amounts of income or educational status—are not applicable or suitable. As such, validation of these scales in non-industrial societies represents an important avenue for future research. While a comparison between MacArthur ladder responses and explicit social comparison questions can help us better understand the factors that may be influencing SSS judgments, much more is needed in way of validation and reliability of these scales across societies. We hope this work is the first step in many in broadening our understanding of how subjective social status develops. Another limitation of this work is that the MacArthur ladder question always preceded the social comparison questions. This may have influenced how children answered the follow-up questions, and further work should systematically counterbalance these questions. Additionally, as our participant pool in other countries is often limited by hard-to-control factors such as which families want to participate and how many children of each age live in the communities we visit, we do not have a uniform age distribution. This may have reduced our power to detect age effects. A third limitation is that we were only able to assess test-retest reliability for one population in India. It’s possible that the pattern of reliability we observe among Indian children does not extend to all populations. These limitations aside, at the broadest level, our findings support the cautious use of the MacArthur ladder across a range of diverse cultures and in children younger than previously investigated. They also highlight the importance of cross-cultural and developmental work for better understanding how the sociocultural environment can shape conceptions of social status.

## References

[1] Operario D, Adler NE, Williams DR. Subjective social status: Reliability and predictive utility for global health. Psychology & Health. 2004;19: 237–246.

[2] Adler NE, Boyce T, Chesney MA, Cohen S, Folkman S, Kahn RL, et al Socioeconomic status and health: the challenge of the gradient. American psychologist. 1994;49: 15 10.1037//0003-066x.49.1.15 8122813

[3] Chen E, Paterson LQ. Neighborhood, family, and subjective socioeconomic status: How do they relate to adolescent health? Health Psychology. 2006;25: 704 10.1037/0278-6133.25.6.704 17100499

[4] Demakakos P, Nazroo J, Breeze E, Marmot M. Socioeconomic status and health: the role of subjective social status. Social science & medicine. 2008;67: 330–340. 18440111 10.1016/j.socscimed.2008.03.038 PMC2547480

[5] Adler NE, Boyce WT, Chesney MA, Folkman S, Syme SL. Socioeconomic inequalities in health: no easy solution. Jama. 1993;269: 3140–3145. 8505817

[6] Cohen S, Alper CM, Doyle WJ, Adler N, Treanor JJ, Turner RB. Objective and subjective socioeconomic status and susceptibility to the common cold. Health Psychology. 2008;27: 268 10.1037/0278-6133.27.2.268 18377146

[7] Kraus MW, Piff PK, Keltner D. Social class, sense of control, and social explanation. Journal of personality and social psychology. 2009;97: 992 10.1037/a0016357 19968415

[8] Davis JA. Status symbols and the measurement of status perception. Sociometry. 1956;19: 154–165.

[9] Quon EC, McGrath JJ. Subjective socioeconomic status and adolescent health: a meta-analysis. Health Psychology. 2014;33: 433 10.1037/a0033716 24245837 PMC5756083

[10] Hoebel J, Müters S, Kuntz B, Lange C, Lampert T. Measuring subjective social status in health research with a German version of the MacArthur Scale. Bundesgesundheitsblatt, Gesundheitsforschung, Gesundheitsschutz. 2015;58: 749–757. 10.1007/s00103-015-2166-x 25986532

[11] Singh-Manoux A, Marmot MG, Adler NE. Does subjective social status predict health and change in health status better than objective status? Psychosomatic medicine. 2005;67: 855–861. 10.1097/01.psy.0000188434.52941.a0 16314589

[12] Baum A, Garofalo J, Yali A. Socioeconomic status and chronic stress: does stress account for SES effects on health? Annals of the New York Academy of Sciences. 1999;896: 131–144. 10.1111/j.1749-6632.1999.tb08111.x 10681894

[13] Adler NE, Epel ES, Castellazzo G, Ickovics JR. Relationship of subjective and objective social status with psychological and physiological functioning: Preliminary data in healthy, White women. Health psychology. 2000;19: 586 10.1037//0278-6133.19.6.586 11129362

[14] Goodman E, Adler NE, Kawachi I, Frazier AL, Huang B, Colditz GA. Adolescents’ Perceptions of Social Status: Development and Evaluation of a New Indicator. Pediatrics. 2001;108: e31–e31. 10.1542/peds.108.2.e31 11483841

[15] Wilkinson RG. Unhealthy societies: the afflictions of inequality Routledge; 2002.

[16] Hu P, Adler NE, Goldman N, Weinstein M, Seeman TE. Relationship between subjective social status and measures of health in older Taiwanese persons. Journal of the American Geriatrics Society. 2005;53: 483–488. 10.1111/j.1532-5415.2005.53169.x 15743294

[17] Sakurai K, Kawakami N, Yamaoka K, Ishikawa H, Hashimoto H. The impact of subjective and objective social status on psychological distress among men and women in Japan. Social Science & Medicine. 2010;70: 1832–1839. 20303205 10.1016/j.socscimed.2010.01.019

[18] Giatti L, do Valle Camelo L, de Castro Rodrigues JF, Barreto SM. Reliability of the MacArthur scale of subjective social status-Brazilian Longitudinal Study of Adult Health (ELSA-Brasil). BMC public health. 2012;12: 1096 10.1186/1471-2458-12-1096 23253581 PMC3545723

[19] Yip W, Adler N. Does social standing affect health and happiness in rural China. 2005.

[20] Fernald LC. Socio-economic status and body mass index in low-income Mexican adults. Social science & medicine. 2007;64: 2030–2042. 17368895 10.1016/j.socscimed.2007.02.002 PMC1924923

[21] Goodman E, Huang B, Schafer-Kalkhoff T, Adler NE. Perceived socioeconomic status: a new type of identity that influences adolescents’ self-rated health. Journal of Adolescent Health. 2007;41: 479–487. 10.1016/j.jadohealth.2007.05.020 17950168 PMC2204090

[22] Piko BF, Fitzpatrick KM. Socioeconomic status, psychosocial health and health behaviours among Hungarian adolescents. The European Journal of Public Health. 2007;17: 353–360. 10.1093/eurpub/ckl257 17130141

[23] Ritterman ML, Fernald LC, Ozer EJ, Adler NE, Gutierrez JP, Syme SL. Objective and subjective social class gradients for substance use among Mexican adolescents. Social science & medicine. 2009;68: 1843–1851. 19342140 10.1016/j.socscimed.2009.02.048

[24] Friestad C, Klepp K-I. Socioeconomic status and health behaviour patterns through adolescence: Results from a prospective cohort study in Norway. The European Journal of Public Health. 2006;16: 41–47. 10.1093/eurpub/cki051 16446300

[25] Potter BK, Speechley KN, Koval JJ, Gutmanis IA, Campbell MK, Manuel D. Socioeconomic status and non-fatal injuries among Canadian adolescents: variations across SES and injury measures. BMC public health. 2005;5: 132 10.1186/1471-2458-5-132 16343342 PMC1334204

[26] Page RM, Suwanteerangkul J. Self‐rated health, psychosocial functioning, and health‐related behavior among Thai adolescents. Pediatrics International. 2009;51: 120–125. 10.1111/j.1442-200X.2008.02660.x 19371291

[27] Salonna F, van Dijk JP, Geckova AM, Sleskova M, Groothoff JW, Reijneveld SA. Social inequalities in changes in health-related behaviour among Slovak adolescents aged between 15 and 19: a longitudinal study. BMC Public Health. 2008;8: 57 10.1186/1471-2458-8-57 18269739 PMC2275256

[28] Nielsen M, Haun D, Kärtner J, Legare CH. The persistent sampling bias in developmental psychology: A call to action. Journal of Experimental Child Psychology. 2017;162: 31–38. 10.1016/j.jecp.2017.04.017 28575664 PMC10675994

[29] Henrich J, Heine SJ, Norenzayan A. The weirdest people in the world? Behav Brain Sci. 2010;33: 61–135. 10.1017/S0140525X0999152X 20550733

[30] Lu F. Integration into the Market among Indigenous Peoples: A Cross‐Cultural Perspective from the Ecuadorian Amazon. Current Anthropology. 2007;48: 593–602. 10.1086/519806

[31] Henrich J, Ensminger J, McElreath R, Barr A, Barrett C, Bolyanatz A, et al Markets, Religion, Community Size, and the Evolution of Fairness and Punishment. Science. 2010;327: 1480–1484. 10.1126/science.1182238 20299588

[32] Urlacher SS, Liebert MA, Josh Snodgrass J, Blackwell AD, Cepon-Robins TJ, Gildner TE, et al Heterogeneous effects of market integration on sub-adult body size and nutritional status among the Shuar of Amazonian Ecuador. Annals of human biology. 2016;43: 316–329. 10.1080/03014460.2016.1192219 27230632 PMC4992548

[33] Liebert MA, Snodgrass JJ, Madimenos FC, Cepon TJ, Blackwell AD, Sugiyama LS. Implications of market integration for cardiovascular and metabolic health among an indigenous Amazonian Ecuadorian population. Annals of human biology. 2013;40: 228–242. 10.3109/03014460.2012.759621 23388068

[34] Urlacher SS, Blackwell AD, Liebert MA, Madimenos FC, Cepon‐Robins TJ, Gildner TE, et al Physical growth of the shuar: Height, weight, and BMI references for an indigenous amazonian population. American Journal of Human Biology. 2016;28: 16–30. 10.1002/ajhb.22747 26126793 PMC4696921

[35] Cepon-Robins TJ, Liebert MA, Gildner TE, Urlacher SS, Colehour AM, Snodgrass JJ, et al Soil-transmitted helminth prevalence and infection intensity among geographically and economically distinct Shuar communities in the Ecuadorian Amazon. Journal of Parasitology. 2014. 10.1645/13-383.1 24865410

[36] Madimenos FC, Snodgrass JJ, Liebert MA, Cepon TJ, Sugiyama LS. Reproductive effects on skeletal health in Shuar women of Amazonian Ecuador: A life history perspective. American Journal of Human Biology. 2012;24: 841–852. 10.1002/ajhb.22329 23015457

[37] Singh-Manoux A, Adler NE, Marmot MG. Subjective social status: its determinants and its association with measures of ill-health in the Whitehall II study. Social science & medicine. 2003;56: 1321–1333. 12600368 10.1016/s0277-9536(02)00131-4

[38] Amir D, Jordan MR, McAuliffe K, Valeggia CR, Sugiyama LS, Bribiescas RG, et al The developmental origins of risk and time preferences across diverse societies. Journal of Experimental Psychology: General. 2019. 10.1037/xge0000675 31512902

[39] US Census Bureau. Profile of general population and housing characteristics: 2010 demographic profile data American Factfinder 2010.

[40] Berube A, Holmes N. City and Metropolitan Inequality on the Rise, Driven by Declining Incomes. Brookings Institution, 1 2016;14.

[41] Kolko J. Normal America is not a small town of white people. Fivethirtyeight com. 2016;28.

[42] Dunham Y, Srinivasan M, Dotsch R, Barner D. Religion insulates ingroup evaluations: the development of intergroup attitudes in India. Developmental Science. 2014;17: 311–319. 10.1111/desc.12105 24205988

[43] Srinivasan M, Dunham Y, Hicks CM, Barner D. Do attitudes toward societal structure predict beliefs about free will and achievement? Evidence from the Indian caste system. Developmental science. 2016;19: 109–125. 10.1111/desc.12294 25754516

[44] Valeggia CR, Burke KM, Fernandez-Duque E. Nutritional status and socioeconomic change among Toba and Wichí populations of the Argentinean Chaco. Economics & Human Biology. 2010;8: 100–110. 19959406 10.1016/j.ehb.2009.11.001 PMC3470426

[45] Lagranja ES, Valeggia CR, Navarro A. Prácticas alimentarias y actividad física en adultos de una población Toba de la provincia de Formosa, Argentina. Diaeta. 2014;32: 35–41.

[46] Lagranja ES, Phojanakong P, Navarro A, Valeggia CR. Indigenous populations in transition: an evaluation of metabolic syndrome and its associated factors among the Toba of northern Argentina. Annals of human biology. 2015;42: 84–90. 10.3109/03014460.2014.932008 25004443 PMC4428908

[47] Bove RB, Valeggia CR, Ellison PT. Girl helpers and time allocation of nursing women among the Toba of Argentina. Human Nature. 2002;13: 457–472. 10.1007/s12110-002-1003-8 26193090

[48] de la Iglesia HO, Fernández-Duque E, Golombek DA, Lanza N, Duffy JF, Czeisler CA, et al Access to electric light is associated with shorter sleep duration in a traditionally hunter-gatherer community. Journal of biological rhythms. 2015;30: 342–350. 10.1177/0748730415590702 26092820 PMC5320422

[49] Barrett HC, Haley KJ. Economic game behavior among the Shuar. Experimenting with social norms: Fairness and punishment in cross-cultural perspective. 2014; 259–274.

